# Cognitive, motor, and visual development in healthy children in the first 42 months of life

**DOI:** 10.5935/0004-2749.20210068

**Published:** 2021

**Authors:** Ana Carla Ramos Vieira da Costa, Nívea Nunes Ferraz, Adriana Berezovsky

**Affiliations:** 1 Departamento de Oftalmologia e Ciências Visuais, Escola Paulista de Medicina, Universidade Federal de São Paulo, São Paulo, SP, Brasil

**Keywords:** Child development, Visual acuity, Cognition, Motor skill, Vision disorders, Neuropsychological tests, Child, Desenvolvimento infantil, Acuidade visual, Cognição, Destreza motora, Transtornos da visão, Testes neuropsicológicos, Criança

## Abstract

**Purpose:**

The Bayley Scales of Infant and Toddler Development-Third Edition (Bayley-III) is a tool for measuring the developmental status of children, including cognitive and motor functioning, in the first three years of life. This study aims to evaluate the correlation between grating visual acuity and visual functionality in healthy children using the Bayley-III.

**Methods:**

Binocular grating visual acuity was measured using Teller Acuity Cards followed by the Bayley-III in healthy children aged 1-42 months. Visual acuity (logMAR) and Bayley-III scores for both cognitive and motor (gross and fine) skills were compared.

**Results:**

Forty children (20 boys) aged 1.2-42.1 months were tested. Their mean visual acuity was 0.39 ± 0.27 logMAR, which was within the normal age limits for all children. There was a strong and significant negative correlation between visual acuity and age (r=-0.83, p<0.001). The mean cognitive raw data score was 49.92 ± 18.93 points, with a strong and significant positive correlation between cognitive score and age (r=0.81, p<0.001). The mean gross motor score was 41.72 ± 16.23 points, with a strong and significant positive correlation between gross motor score and age (r=0.75, p<0.001). The mean fine motor score was 39.75 ± 14.63 points, with a strong and significant positive correlation between fine motor score and age (r=0.77, p<0.001). Multiple linear regression demonstrated that older age and better visual acuity were significantly associated with higher Bayley-III scores.

**Conclusions:**

This study found a high correlation between grating visual acuity measured using Teller Acuity Cards and cognitive and motor scores measured using the Bayley-III in healthy children, demonstrating that the Bayley-III might be a useful tool for assessing the repercussions of visual impairment on the cognitive and motor development of young children.

## Abstract (pt)

**Objetivo:**

A Escala Bayley de Desenvolvimento Infantil (Bayley-III) é uma ferramenta que avalia o desenvolvimento de crianças nos 3 primeiros anos de vida, incluindo os domínios cognitivo e motor. Este estudo tem como objetivo correlacionar a acuidade visual de grades e a funcionalidade visual em crianças saudáveis usando a Bayley-III.

**Métodos:**

A acuidade visual binocular de grades foi medida usando o teste dos Cartões de Acuidade de Teller seguido pela Bayley-III em crianças saudáveis com idade entre 1-42 meses. Os escores da acuidade visual (logMAR) e da Bayley-III para habilidades cognitivas e motoras (grossa e fina) foram comparados.

**Resultados:**

Um grupo de 40 crianças (20 meninos) com idades entre 1,2-42,1 meses foi testado e a média da acuidade visual foi de 0,39 ± 0,27 logMAR, sendo que todas estavam dentro dos limites normais para a idade. Houve uma forte correlação negativa e significante entre acuidade visual e idade (r=-0,83; p<0,001). A média do escore cognitivo foi de 49,92 ± 18,93 pontos, com forte correlação positiva e significante entre o escore cognitivo e a idade (r=0,81; p<0,001). A média do escore motor grosso foi de 41,72 ± 16,23 pontos, com forte correlação positiva e significante entre o escore motor grosso e a idade (r=0,75; p<0,001). A média do escore motor fino foi de 39,75 ± 14,63 pontos, com uma forte correlação positiva e significante entre o escore motor fino e a idade (r=0,77; p<0,001). A regressão linear múltipla mostrou que maior idade e melhor acuidade visual foram significantemente associadas à escores cognitivo e motor mais altos.

**Conclusões:**

Neste estudo foi encontrada alta correlação entre a acuidade visual de grades medida pelos cartões de acuidade de Teller e os escores cogninitivo e motor medidos pela Bayley-III em crianças saudáveis. A Bayley-III pode ser uma ferramenta útil para avaliar a repercussão da deficiência visual no desenvolvimento cognitivo e motor de crianças.

## INTRODUCTION

Neuropsychomotor development is characterized by continuous changes in motor, psychological, and cognitive behavior throughout the life cycle of an individual^(1)^. The acquisition of motor skills occurs in steps and follows a sequence of behaviors based on the age of the child, including creeping (after 2 months), sitting (6-7 months), crawling (8-10 months), unsupported standing (12-13 months), and walking (14-18 months)^(2)^.

The formation of emotional bonds also occurs over this long process of development and is triggered by basic daily activities, such as self-care tasks, as well by more complex activities of daily living, including social life and leisure^(3,4)^. Visual experience during childhood plays a fundamental role in the formation and maturation of cortical circuits as well as in the mental, motor, and emotional development of the child^(5,6)^.

The Bayley Scales of Infant and Toddler Development-Third Edition (Bayley-III) is a tool used in the assessment of a child’s neuropsychomotor development. The newest version was standardized in 2006 and comprises five distinct domains: cognitive, motor (divided into gross and fine), language (divided into communicative and expressive), emotional, and social adaptive behaviors. During the administration of the Bayley-III, an examiner asks children to perform various tasks that include some visual stimuli to assess their cognitive, motor (gross and fine), and language skills^(7,8)^.

The first year of life is critical for cognitive, motor, and visual development, and deprivation of adequate stimulation during this phase can result in irreversible functional damage^(9,10)^. This initial period is called the critical period and is essential for the formation of neural circuits necessary for vision^(11,12)^. In infants, preverbal infants, or patients with communication disorders, visual acuity (VA) is measured independent of verbal response and is evaluated clinically using psychophysical methods, such as the Teller Acuity Cards (TAC) test, or using electrophysiological methods, such as sweep visually evoked potentials^(13-16)^.

This study aims to evaluate the correlation between grating VA and visual functionality in healthy children using the Bayley-III.

## METHODS

This observational cross-sectional study was conducted between June 2016 and December 2017 at the Department of Ophthalmology and Visual Sciences, School of Medicine/Federal University of São Paulo (UNIFESP). The study followed the basic principles of the Declaration of Helsinki and was approved by the Research Ethics Committee of UNIFESP. Full-term children of both genders (aged 1-42 months) who were free from disease and/or neurological or ocular disorders and with normal ocular motility were included in this study after the parents signed the informed consent form. Children with strabismus and nystagmus as well as those with binocular grating VA below the normal limits for the age as measured by the TAC were excluded.

### Procedures

The examinations comprised measurement of binocular grating VA using the TAC and applying the Bay ley-III after the certification of ocular alignment and normal vi sual behavior by assessing ocular motility, quality of fixation, and ability to follow light and objects.

### TAC test

The examiner, accompanied by an assistant, performed the TAC test in the binocular condition, 55 cm away from the child. The binocular grating VA threshold was defined as the arithmetic mean of the card values corresponding to six reversals, and the results were compared with the normative values of VA for age^(17)^.

### Bayley-III

The Bayley-III for cognitive (91 items), gross motor (72 items), and fine motor (66 items) domains was sequentially applied binocularly following the manufacturer’s instructions^(8)^. The starting point of the test was determined according to the child’s age in months and days. For each test, a task that was successfully performed by the child was scored as 1, whereas a score of 0 was recorded when the child failed to perform the task after three attempts. At the beginning of the test, a sum of three consecutive points was required from the starting point; however, in cases in which a score of 0 was obtained in any of the first three items, the test was started from the immediately preceding starting point. When a score of 0 was obtained on five consecutive items, the test was discontinued. For each domain of the Bayley-III, the gross score was calculated from the arithmetic sum of the score obtained in the test, adding to the score the value of the total score corresponding to the starting point. The time of administration varied with age, corresponding to approximately 50 minutes for children 1-12 months of age and 90 minutes for children 13-42 months of age.

### Statistical analysis

Data were analyzed using Stata Data Analysis and Statistical Software version 12. The use of parametric or nonparametric statistical models was guided by the symmetry and kurtosis of the data in addition to the Shapiro-Wilk normality test. When there was no normal distribution of variables, nonparametric tests were used. The level of statistical significance was considered as *p*≤0.05 with a two-tailed rejection region.

Correlations between the thresholds of VA (logMAR) obtained using the TAC and the scores of the cognitive, gross motor, and fine motor domains of the Bayley-III were analyzed. The results were evaluated in terms of gender, age, and parents’ level of schooling. Statistical models included the Student *t* test, Mann-Whitney *U* test, Pearson correlation, simple linear regression, and multiple linear regression.

## RESULTS

A total of 43 children were evaluated. One child had visual impairment measured by the TAC, whereas two children showed cognitive and gross motor changes after application of the Bayley-III, which were compatible with gait alteration, despite a normal VA threshold in TAC. Therefore, the data of these three children from the initial sample were discarded. Based on the inclusion criteria, 40 children (20 boys) aged 1.25-42.11 months (mean and median of 16.87 ± 10.34 and 14.51 months, respectively) were eligible for this study. There was no statistically significant difference in the age of the children distributed by gender.

VA ranged from 0.01-1.06 logMAR (mean= 0.39 ± 0.28 logMAR, median = 0.37 logMAR). There was a strong and significantly negative correlation between VA and age (Pearson *r*=-0.83, *p*<0.001). Student *t* test demonstrated that VA was statistically worse (*t*=-2.22487; *p*=0.0304) in male children (0.49 ± 0.26 logMAR) than in female children (0.30 ± 0.27 logMAR); however, after adjusting for gender (*p*=0.204) in the multiple linear regression, VA was associated only with age (*p*<0.001).

Table 1 shows the distribution of cognitive, gross motor, and fine motor scores of the 40 children who were examined. A strong and significantly positive correlation (Pearson *p*<0.001) was observed between age and cognitive (*r*=0.81), gross motor, (*r*=0.75), and fine motor (*r*=0.77) scores. Simple linear regression demonstrated that the expected increase in cognitive score was 1.49 points (*p*<0.001), in gross motor score was 1.18 points (*p*<0.001), and in fine motor score was 1.09 points (*p*<0.001) in each month of life. There was no statistically significant difference in the scores among the children distributed by gender.

**Table 1:** Distribution of cognitive, gross motor, and fine motor scores by gender

		Bayley-111 domains		
		Cognitive	Gross motor	Fine motor
Gender	n	Mean (SD)	Mean (SD)	Mean (SD)
Female	20	54.95 (19.43)	44.40(16.83)	41.60(14.92)
Male	20	44.90(17.46)	39.05 (15.59)	37.90(14.48)
Total	40	49.93 (18.93)	41.73 (16.23)	39.75 (14.63)

n= number of children; SD= standard deviation.

Multiple linear regression (Table 2) indicated a statistically significant association between cognitive score and age, VA, and parents’ schooling. The expected increase in the cognitive score was 1.01 points (*p*=0.001) in each month of life and 2.18 points (*p*=0.049) for each line of improvement in VA; for children whose parents had a high school or less level of schooling, the expected reduction in score was 7.84 points (*p*=0.020), after adjusting for other variables. Motor scores were statistically significantly associated with age and VA, with an expected increase of 0.63 points (*p*=0.036) in gross motor score and 0.67 points (*p*=0.008) in fine motor score in each month of life, and for each line of improvement in VA, an increase of 2.71 points (*p*=0.019) and 2.22 points (*p*=0.020) respectively, after adjusting for other variables.

**Table 2:** Relationship between scores, age, gender, VA, and parents’ schooling

	Bayley-III domains					
	Cognitive		Gross motor		Fine motor	
	Coefficient	*p*	Coefficient	*p*	Coefficient	*P*
Age	1.012	0.001^*^	0.626	0.036^*^	0.665	0.008^*^
Gender						
Female	Reference		Reference		Reference	
Male	0.168	0.961	3.289	0.355	4.464	0.131
VA (lines)	-2.183	0.049^*^	-2.711	0.019^*^	-2.215	0.020^*^
Parents’ schooling						
College/university completed Reference	Reference		Reference		Reference	
High school or less	-7.841	0.020^*^	-0.402	0.904	-4.870	0.083

VA= binocular grating visual acuity; *p*= statistical significance.

## DISCUSSION

This study showed a significant improvement of VA from 1.06 logMAR (20/230) at 1.25 months to 0.01 logMAR (20/20) at 42.11 months of age, in agreement with previous studies^(15,17-19)^. Grating VA normative data in infancy guide the determination of vision impairment, and it plays an important role in the diagnosis and treatment of visual deficits in eye conditions, including refractive errors, amblyopia, and cerebral visual impairment^(20)^. In the present study, VA was statistically better on average in girls (0.30 logMAR) than in boys (0.49 logMAR); however, the mean age of girls (19.71 months) was higher on average than in boys (14.02 months). These re sults differ from the findings observed in previous studies^(17,18)^. In addition, various studies about the anatomical development of visual pathways in healthy individuals suggest that maturation is accelerated in girls^(21-23)^.

A previous study evaluated a population of 646 healthy children aged 0.5-36 months to determine the normative values for VA using the TAC. VA ranged from 0.66 (1.66 logMAR, 20/909) to 17.82 cycles/degree (1.53 logMAR, 20/34), with no differences based on gender or ethnicity. Furthermore, the study included normality data for monocular VA, which ranged from 2.31 (1.11 logMAR, 20/260) at 2 months to 14.98 cycles/degree (0.30 logMAR, 20/40) at 36 months of age. In another similar study comprised of 460 children (aged 1-4 years) with normal visual development, monocular acuity ranged between 0.94 (1.50 logMAR, 20/638) and 24.81 cycles/degree (0.08 logMAR, 20/24) and did not differ significantly according to gender, ethnicity, schooling, income, or parents’ age^(18)^.

With the use of the Bayley-III in our study, we found a significant improvement in cognitive score, which increased from 11 points at 1.25 months to 65 points at 42.11 months. A similar improvement also observed for gross motor score, which improved from 12 to 57 points, as well as for fine motor score, which improved from 4 to 49 points. This improvement with age is in agreement with the findings of previous studies in healthy children^(24-28)^. However, previous normative studies of cognitive domains (fine and gross motors) of the Bayley-III did not take into account the distribution by sex.

Table 3 summarizes the findings of different studies that have used the Bayley-III to analyze the development of healthy children in Brazil and other countries. In comparing the studies by Andrade et al.^(3)^ and Santos et al.^(24)^, it can be observed that the children’s cognitive scores were higher than that found in the present study. This can probably be explained by the fact that these studies used composite scores of the previous version of the Bayley scale (second version), whereas our study used the Bayley-III (last version) gross score. Composite scores are part of a group of derived scores and are expressed as a function of measures of central tendency and variability, such as mean, standard deviation (SD), percentile, and confidence interval. According to the Bayley-III manual^(8)^, a score of 100 in any domain is defined as the average performance of a given normal group, with SD equal to zero. Therefore, a score higher than found in the current study was expected. As for the study by Ballot et al.^(25)^ in South Africa, although the authors used the last version of the Bayley-III, composite score analysis was performed for cognitive, gross motor, and fine motor domains.

**Table 3:** Comparison of cognitive, gross motor, and fine motor scores of previous studies

Author	Country		Year	N	Age (months)		Cognitive score	Gross motor score		Fine motor score
Andrade et al.^(3)^	Brazil		2005	350	17-42		96.3 ± 11.02	N.A.		N.A.
Santos et al.^(25)^	Brazil		2008	320	20-42		96.84 ± 10.14	N.A.		N.A.
Visser et al.^(27)^	Netherlands		2013	41	1.18-44.20		60.00 ± 21.60	47.50 ±18.10		39.00 ± 14.70
Madaschi et al.^(28)^	Brazil		2016	207	12-42		64.78 ± 10.85	48.49 ± 8.68		33.43 ± 9.20
Ballot et al.^(26)^	South Africa		2017	74	9-20		92.20	98.80		
							(95% CI 89.4-95.0)	(95% CI 96.8-101.0)		
Present study	Brazil		2018	43	1.25-42.11		49.93 ± 18.93	41.73 ± 16.23 39.75 ± 14.63		

N= Number of participants; N.A.= Not applicable.

Table 3 shows that the mean cognitive and gross motor scores were higher in the studies conducted by Visser et al.^(26)^ and Madaschi et al.^(27)^ than in the present study. It is worth mentioning that the age of the subjects in the study by Madaschi et al.^(27)^ was 12-42 months whereas it was 1.18-44.20 months in the study by Visser et al.^(27)^, with an average of 24.50 months. However, the mean subject age was 16.87 months in our study.

The present study demonstrated a statistically significant association between cognitive score and parents’ schooling, as demonstrated by multiple linear regression. These results are in agreement with the findings of a previous study of Santos and Damasceno^(28)^. For children whose parents had a high school or less level of education, the expected reduction in the cognitive score was 7.84 points, showing a relationship between better quality of the stimuli offered to the child and better parental schooling.

In a study in Italy^(29)^ that used the TAC to evaluate VA in the first 2 years of life in healthy children, the authors found that cognitive and motor development as assessed by the Bayley-II were age compatible. However, this study did not correlate VA data with the obtained scores. An extensive bibliographical search conducted among scientific databases returned no results for previous normative studies that performed this analysis using the Bayley-III. As expected, in the multiple linear regression, the current study showed that older age and better VA were significantly associated with higher Bayley-III scores (cognitive, gross motor, and fine motor).

The quantitative evaluation of VA associated with the Bayley-III may broaden our understanding of neuropsychomotor development in the first years of life. This could assist in the therapeutic planning of visual habilitation/rehabilitation and the establishment of educational strategies, which would contribute to improvements in visual behavior, motor skills, and social interaction, positively affecting the child’s quality of life. This study can provide a basis for further research comprising larger samples for the study of quantitative measures of VA as well as cognitive and motor performance in children with ocular disorders.

In conclusion, this study found a strong correlation between grating acuity threshold as measured by the TAC and cognitive and motor scores as measured by the Bayley-III in this cohort of healthy children. To better analyze the impact of visual impairment in developing children, further studies are needed that include pediatric patients with a range of ocular conditions, to enable a more thorough investigation of the usefulness of the Bayley-III test in children with visual impairment, neurological disorders, and other developmental conditions.
